# Supplementary material for: Comprehensive Cytokine Profiling of Patients with COVID-19 Receiving Tocilizumab Therapy
Source: Int J Mol Sci. 2022 Jul 19;23(14):7937. doi: 10.3390/ijms23147937 (PMC9316906; doi:10.3390/ijms23147937)
Supplement: Supplementary file 1 [file ijms-23-07937-s001.zip › ijms-1802075-supplementary.pdf]

Supplementary material

Table S1. Cytokine concentrations in clusters 1 and 2.
Data are presented as log2 of the normalized fluorescence values with median and interquartile range [Q25;Q75]. *P*-values are presented according to the Mann-Whitney test and with the Benjamini-Hochberg FDR correction for adjusted *p*-values.
N.Fl. - normalized fluorescence intensity; BH - Benjamini-Hochberg.

	Cluster 1 median, log2(N.Fl.)	Cluster 1 [Q25;Q75], log2(N.Fl.)	Cluster 2 median, log2(N.Fl.)	Cluster 2 [Q25;Q75], log2(N.Fl.)	Mann-Whitney p-val	Mann-Whitney p-val with BH correction
PDGF-AA	11.7	[11.3;12.2]	12.6	[12.0;13.3]	1.30×10^{-6}	1.60×10^{-6}
PDGF-AB/BB	9.9	[9.6;10.6]	10.9	[10.3;11.7]	1.10×10^{-6}	1.50×10^{-6}
IL-1b	4.4	[4.3;4.5]	4.9	[4.7;5.1]	1.60×10^{-14}	6.50×10^{-13}
IL-10	5.4	[5.0;6.2]	6.2	[5.8;6.8]	9.90×10^{-5}	1.10×10^{-4}
sCD40L	6.9	[6.1;7.5]	8.6	[7.8;9.8]	6.30×10^{-9}	1.20×10^{-8}
MIP-1b	6.8	[6.2;7.1]	7.7	[7.0;8.1]	2.60×10^{-6}	3.30×10^{-6}
IL-6	5.7	[5.2;6.6]	7	[6.2;7.9]	3.70×10^{-8}	5.90×10^{-8}
Fractalkine	4.1	[3.9;4.3]	4.6	[4.4;4.8]	1.00×10^{-9}	2.70×10^{-9}
IL-8	6.3	[6.0;6.7]	7.3	[7.0;7.6]	5.20×10^{-11}	2.30×10^{-10}
MCP-1	11	[10.1;11.4]	11.9	[11.3;12.4]	4.90×10^{-6}	6.00×10^{-6}
TNFa	5.8	[5.5;6.2]	6.3	[6.1;6.5]	4.40×10^{-07}	6.30×10^{-07}
IP-10	12.9	[12.0;13.6]	13.4	[12.9;13.8]	2.30×10^{-02}	2.40×10^{-02}
IL-2	4.5	[4.3;4.6]	4.9	[4.7;5.2]	4.00×10^{-12}	2.70×10^{-11}
MIP-1a	4.5	[4.3;4.6]	5.1	[4.9;5.3]	1.40×10^{-13}	1.90×10^{-12}
IL-3	4.8	[4.4;5.1]	5.4	[5.2;5.8]	1.50×10^{-9}	3.70×10^{-9}
IL-4	4.3	[4.0;4.5]	4.8	[4.5;5.2]	7.80×10^{-10}	2.30×10^{-9}
IL-17A	5	[4.9;5.2]	5.4	[5.2;5.5]	1.30×10^{-8}	2.20×10^{-8}
GM-CSF	4.5	[4.3;4.6]	5.1	[4.9;5.3]	1.20×10^{-12}	1.20×10^{-11}
EGF	5.7	[5.2;6.4]	7.4	[6.4;8.1]	8.20×10^{-9}	1.40×10^{-8}
FGF-2	4	[3.9;4.3]	4.5	[4.3;4.8]	1.80×10^{-9}	4.10×10^{-9}
Eotaxin	6.6	[6.0;7.0]	6.8	[6.5;7.4]	2.40×10^{-02}	2.40×10^{-02}
TGF-a	4.7	[4.5;4.8]	5.1	[5.0;5.4]	5.30×10^{-12}	3.00×10^{-11}
G-CSF	5.4	[5.0;6.1]	5.9	[5.5;6.5]	1.30×10^{-03}	1.40×10^{-03}
Flt-3L	4.6	[4.4;4.9]	4.9	[4.7;5.1]	7.50×10^{-6}	8.80×10^{-6}
IFNa2	4.2	[4.1;4.5]	4.7	[4.5;4.9]	1.50×10^{-07}	2.20×10^{-07}
IFNg	4.5	[4.2;4.7]	5.1	[4.8;5.5]	3.20×10^{-9}	6.10×10^{-9}
GRO	10.6	[9.8;11.2]	11.8	[11.2;12.5]	5.40×10^{-8}	8.30×10^{-8}
MCP-3	4.2	[4.0;4.4]	4.7	[4.5;5.4]	3.80×10^{-10}	1.30×10^{-9}
IL-12p40	4.5	[4.4;4.7]	4.9	[4.7;5.1]	2.80×10^{-9}	5.90×10^{-9}
MDC	8.4	[7.8;8.9]	8.4	[8.0;8.7]	4.70×10^{-01}	4.70×10^{-01}
IL-12p70	4.3	[4.2;4.4]	4.7	[4.5;4.8]	8.00×10^{-10}	2.30×10^{-9}
IL-13	4	[3.8;4.2]	4.5	[4.2;4.7]	2.30×10^{-9}	5.00×10^{-9}
IL-15	5	[4.8;5.2]	5.3	[5.1;5.5]	6.80×10^{-07}	9.40×10^{-07}
IL-1RA	5.5	[4.9;6.3]	6.3	[5.8;6.9]	3.40×10^{-04}	3.80×10^{-04}
IL-1a	4.9	[4.7;5.2]	5.6	[5.3;5.9]	6.10×10^{-11}	2.40×10^{-10}
IL-9	4.5	[4.3;4.6]	5.1	[4.9;5.3]	8.70×10^{-14}	1.70×10^{-12}

**IL-5**	4.4	[4.2;4.5]	4.9	[4.7;5.1]	2.20×10^{-11}	1.10×10^{-10}
**IL-7**	4.2	[4.0;4.5]	4.7	[4.5;4.9]	1.50×10^{-10}	5.30×10^{-10}
**TNFb**	4.4	[4.3;4.6]	4.9	[4.7;5.3]	3.10×10^{-9}	6.10×10^{-9}
**VEGF**	4.3	[4.1;4.5]	5	[4.7;5.4]	2.90×10^{-12}	2.30×10^{-11}

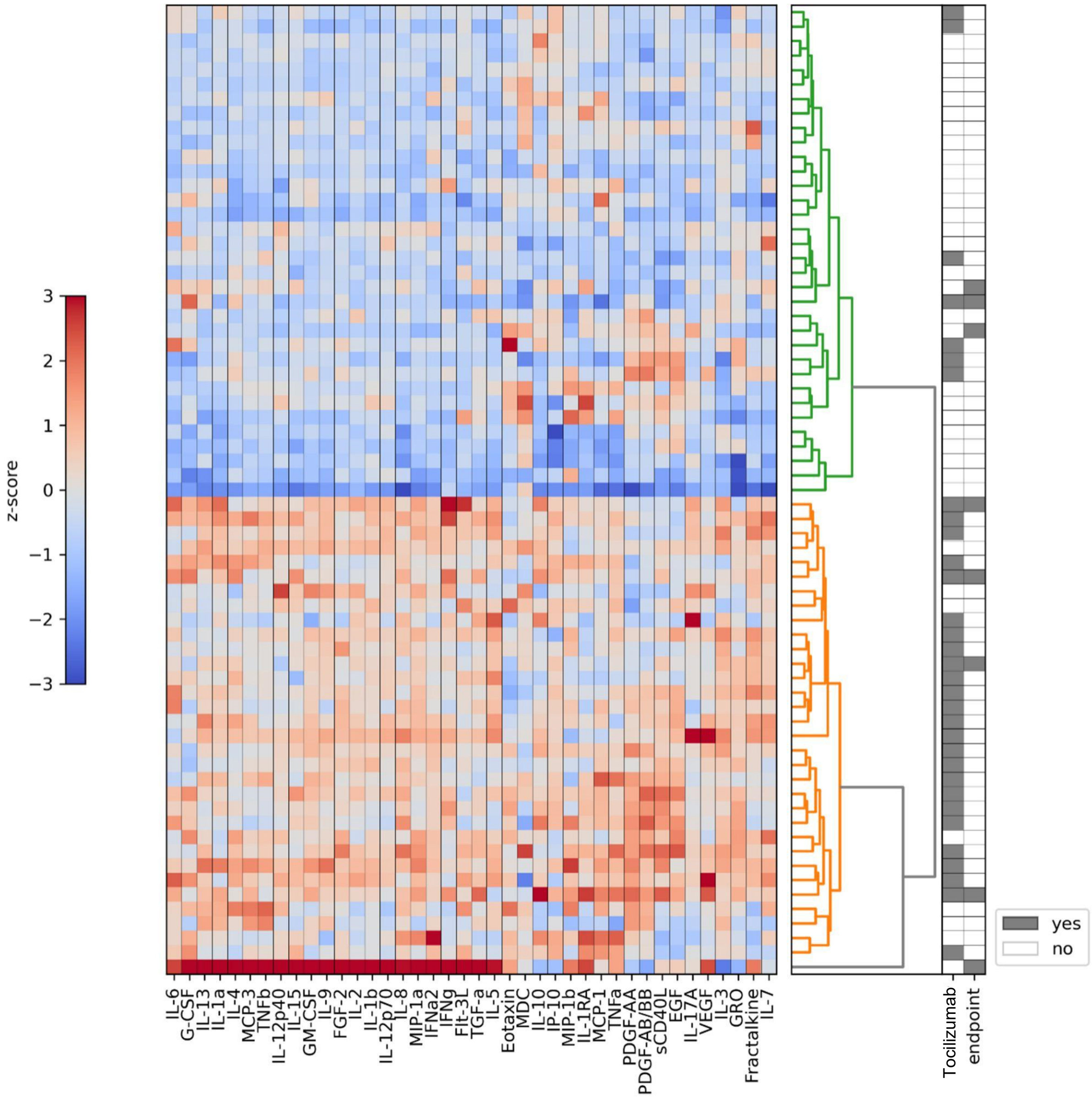

Figure S1. Patients' cytokine clusterization in the non-steroid group at the timepoint 1. Data are presented as log2 of the normalized fluorescence intensity values that were further z-score normalized and used as a basis for hierarchical clustering analysis. Cluster 1 is indicated with the green hierarchical tree, cluster 2 is indicated with the orange hierarchical tree. Tocilizumab indicates application of the immunomodulatory therapy. Endpoint indicates a presence of a combined clinical endpoint which included application of high-flow oxygen therapy or noninvasive/invasive lung ventilation, patient transfer to intensive care, and in-hospital mortality.

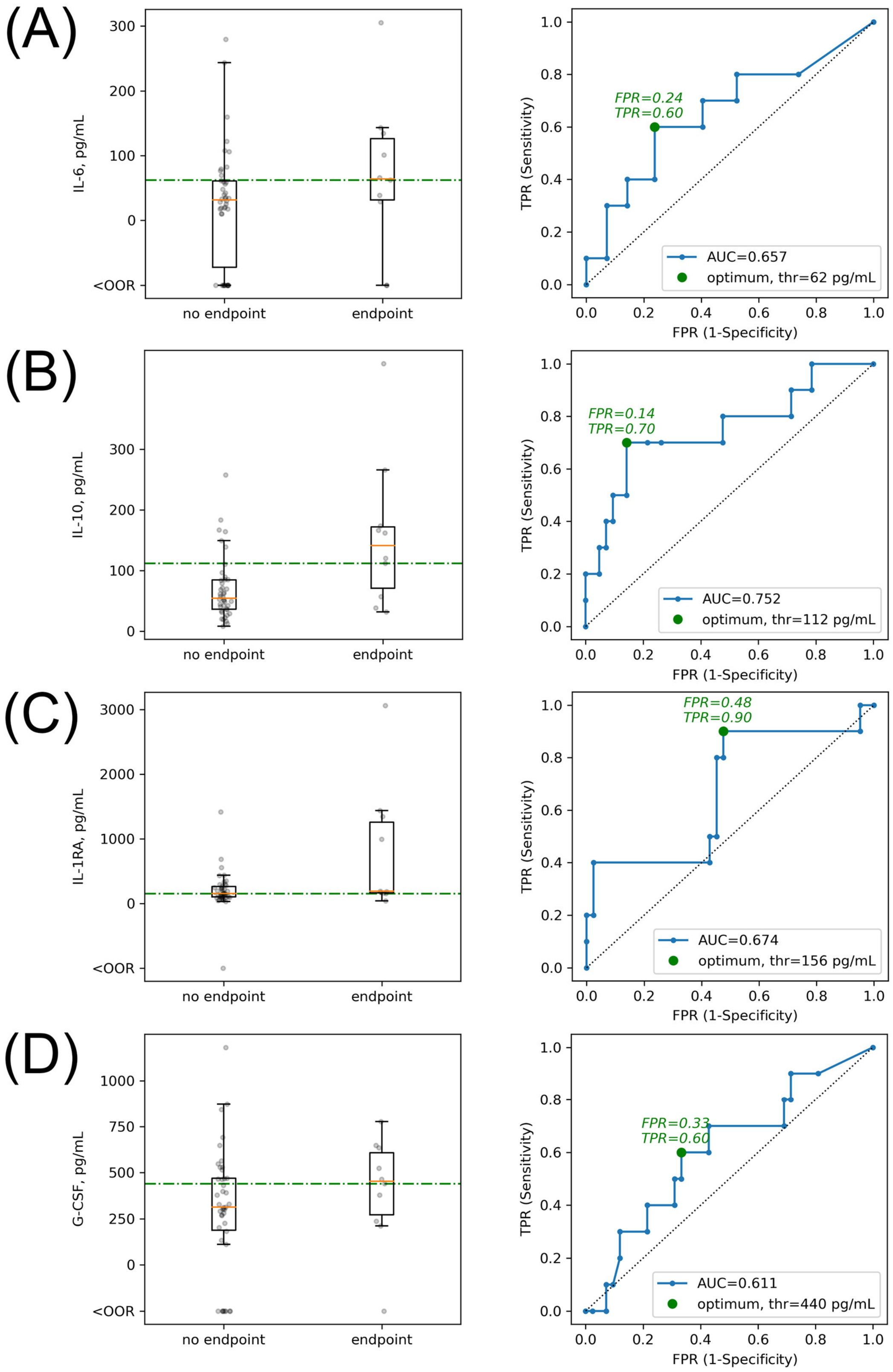

Figure S2. Boxplots for the absolute concentrations and ROC curves for selecting optimal concentration threshold for the cytokines. Presented are 4 cytokines prominently associated with clinical endpoints in patients with tocilizumab therapy. IL-6 (A). IL-10 (B). IL-1RA (C). G-CSF (D). OOR - out of range (< lower limit of detection).

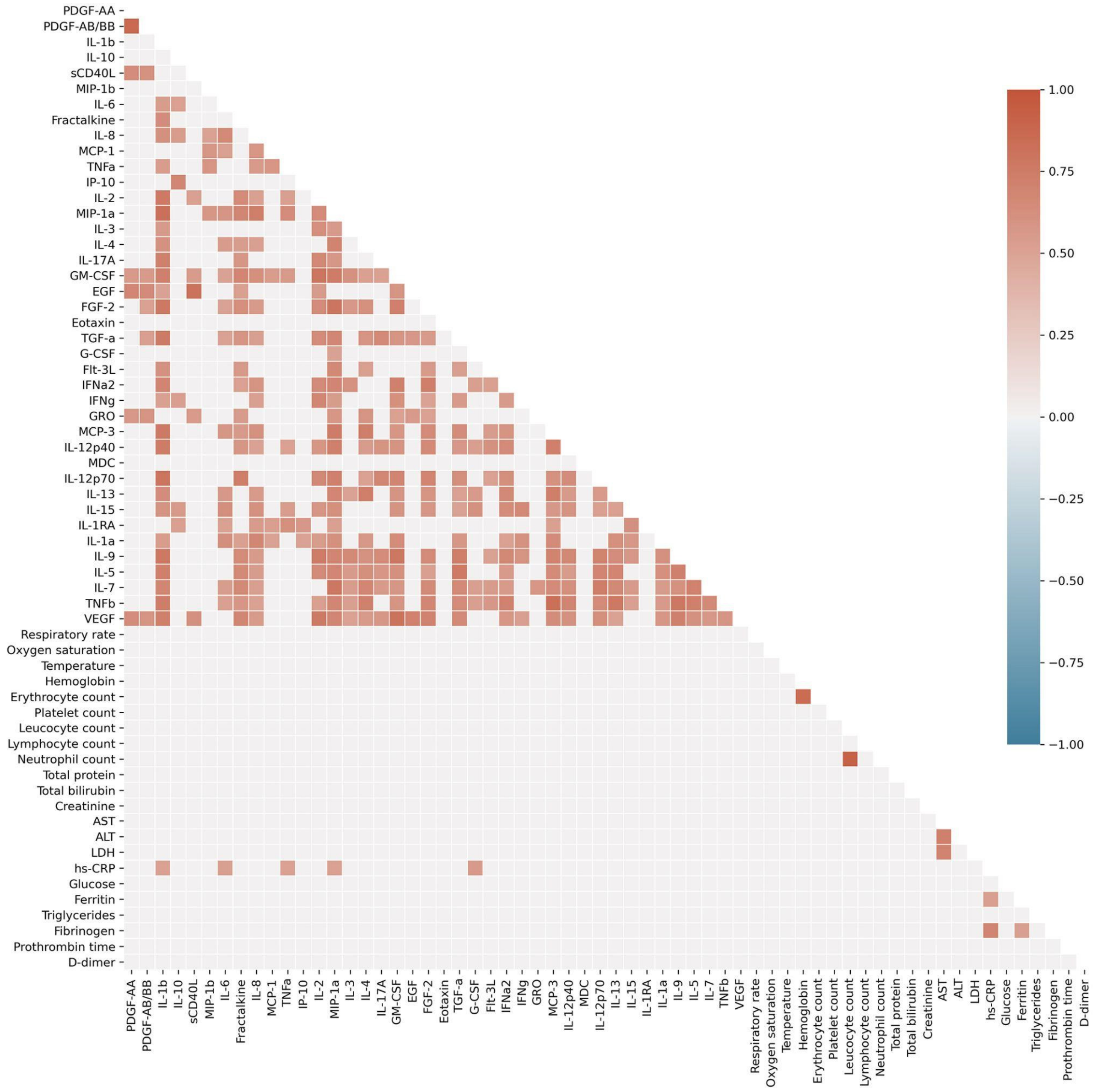

Figure S3. Correlation matrix of cytokines and laboratory parameters.
Data are shown as Spearman correlations between cytokine levels and laboratory test results. Only correlations with coefficient > 0.5 and p-value < 0.05 are shown. For the cytokine levels log2 of the normalized fluorescence values are used.

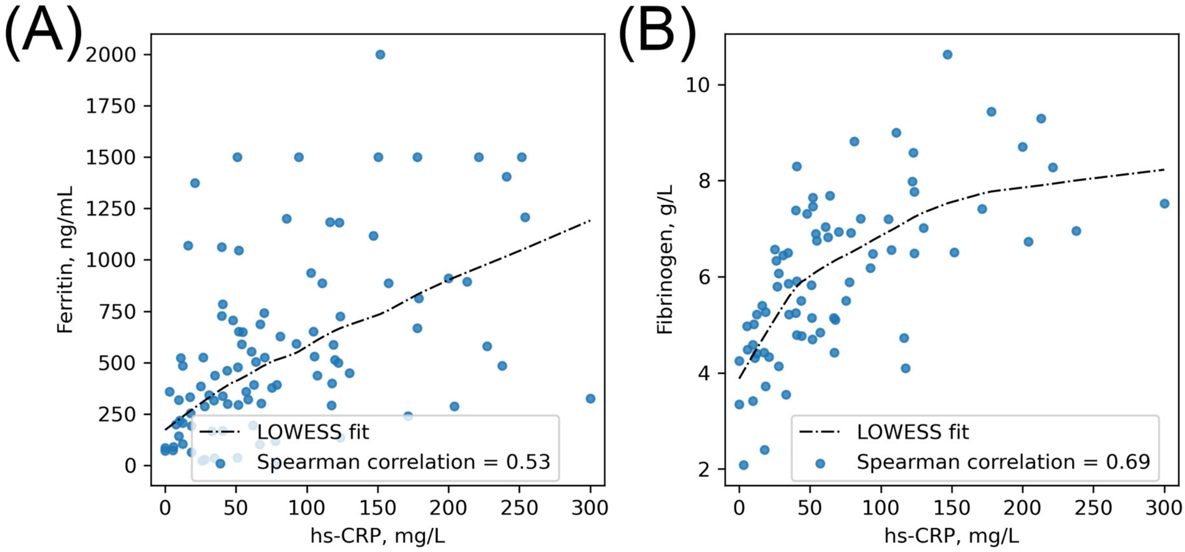

Figure S4. Correlation between hs-CRP and laboratory parameters. Ferritin vs hs-CRP (A). Fibrinogen vs hs-CRP (B). Data are shown as Spearman correlations.

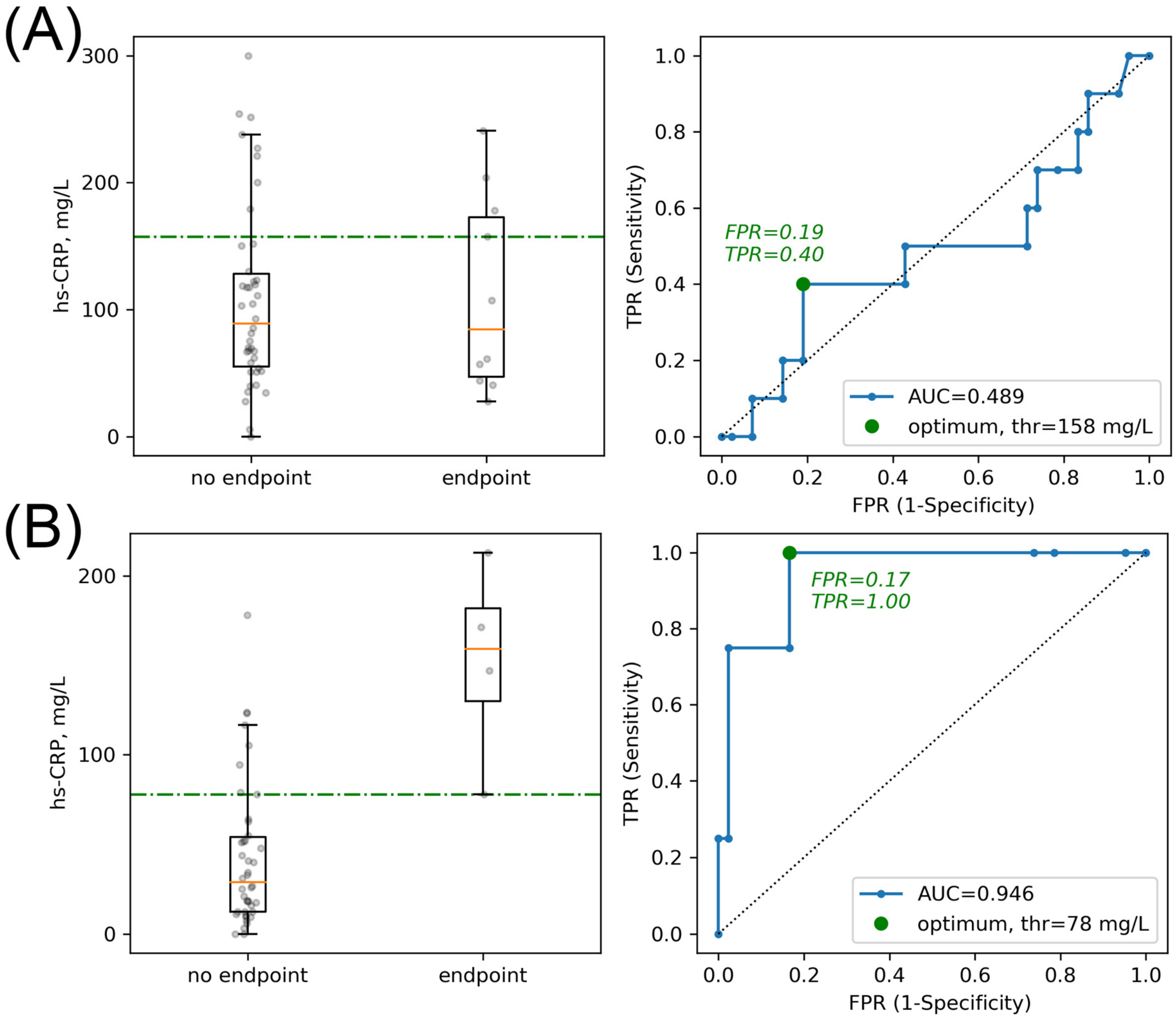

Figure S5. Boxplots for the absolute concentrations and ROC curves for selecting optimal concentration threshold for hs-CRP level. Patients with (A) and without tocilizumab therapy (B).
